# Discontinuous transcription

**DOI:** 10.1080/19491034.2017.1419112

**Published:** 2018-01-30

**Authors:** Evgeny Smirnov, Matúš Hornáček, Tomáš Vacík, Dušan Cmarko, Ivan Raška

**Affiliations:** Institute of Biology and Medical Genetics, First Faculty of Medicine, Charles University and General University Hospital in Prague, Prague, Czech Republic

**Keywords:** Pulsing transcription, bursting, transcriptional fluctuation, discontinuous gene expression, kinetics

## Abstract

Numerous studies based on new single-cell and single-gene techniques show that individual genes can be transcribed in short bursts or pulses accompanied by changes in pulsing frequencies. Since so many examples of such discontinuous or fluctuating transcription have been found from prokaryotes to mammals, it now seems to be a common mode of gene expression. In this review we discuss the occurrence of the transcriptional fluctuations, the techniques used for their detection, their putative causes, kinetic characteristics, and probable physiological significance.

## Introduction

Kinetics may lie at the heart of the mysterious ability of biological molecules to work in concert to regulate gene expression at the whole genome level [1]. Measuring bulk levels of RNA by such methods as Northern blotting, RT-PCR or RNA-Seq gives the impression that transcription is usually continuous, that once started it proceeds at the same rate. But the studies based on new single-cell and single-gene techniques indicate that this is not the case. Perhaps, such terms as pulsing, bursting, or fluctuation describe temporal course of transcription more correctly. In this review we discuss occurrence of transcriptional fluctuations, the techniques used for detection of this pulsatile activation, as well as its supposed causes, dynamic characteristics, and probable physiological significance.

## 1. Transcriptional fluctuation is a common feature of gene expression.

Although the oscillating nature of transcription has drawn quite a bit of attention in the past two decades, the existence of this phenomenon was suggested much earlier. An irregular distribution of the nascent transcripts on the non-ribosomal DNA strands was observed in a study of the chromatin spreads of the Drosophila melanogaster cells [2]. The authors assumed that the gaps separating the series of polymerase complexes might result from interruptions in initiation of transcription.

Transcriptional fluctuation has been discovered in the cells of diverse species ranging from prokaryotes to mammals; in developing embryos as well as in various cell differentiation systems including embryonic stem cells [3–12]. Viral genes and curiously enough also the gene encoding the largest subunit of RNA polymerase II (pol II) exhibit pulse-like expression patterns [13,14]. The discontinuous mode of transcription seems to be very common, perhaps even predominant, at least in mammalian cells [6,7,15].

Not surprisingly, various genes in the same cell display a wide range of transcriptional kinetic behaviour [6,16–19]. In the yeast 1 out of 4 examined genes displayed a clearly pulsing pattern, but for the other three genes, all housekeeping genes, such pattern was not observed [20]. From their data the authors of this study hypothesized that intensive transcription tends to be continuous. This is further supported by the study of the *cyclin D1* gene whose transcription was pulse-like when driven by its own promoter, but became continuous under the powerful *CMV* promoter [18]. Nevertheless, one should bear in mind that transcriptional fluctuations have different kinetic characteristics (see the section 4), and may elude observation, for instance, if the intervals between the successive measurements are not sufficiently short. Besides, some data show that housekeeping genes may also be transcribed in a pulse-like manner [22,23].

Although the discontinuous expression has been studied predominantly in genes transcribed by pol II, the products of RNA polymerase III, which also works under a complex regulation, [24] are also likely to be issued in pulses. On the other hand, it is very difficult to analyze the discontinuous transcriptional activity driven by RNA polymerase I (pol I), since the ribosomal genes transcribed by pol I exist in numerous copies and their expression is usually very intensive. However, the direct measurements of ribosomal RNA production in the entire nucleoli by the label-free confocal Raman microspectrometry showed a pulse-like pattern of the ribosomal DNA transcription [23]. In our work on tumour-derived cells expressing a GFP-RPA43 (a subunit of pol I) fusion protein, we measured the fluorescence signal upon the nucleolar beads, which are likely to represent individual transcriptionally active genes [25–29]. Our data, complemented with the measurements of nascent transcription revealed by the incorporated fluorouridine signal, suggested that the ribosomal genes are also transcribed in pulse-like manner [25].

**Figure 1.**
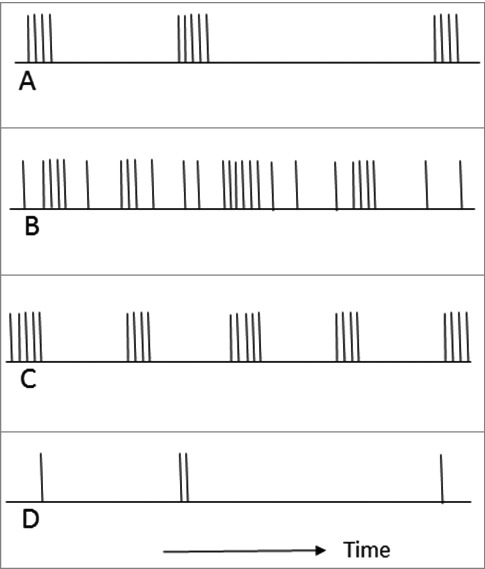
Common patterns of discontinuous transcription. Each vertical line represents one event of RNA synthesis, i.e. one termination. **A**: Typical bursts: irregular and alternated by long intervals of silence. The burst size tends to be constant. **B**: An undulating pattern with rare transcription events between the bursts. **C**: Regular pulsing based on feedback loops. **D**: Rare transcription events.

Transcriptional fluctuation seems to be irregular in most cases (Figures 1A, 1B, and 1D), but it may also appear as bursts separated by periods of inactivity (Figure 1C) [6]. Such regular or oscillatory patterns of gene expression are observed when a gene is involved in a regulatory circuit with a negative feedback [1]. For example, the estradiol receptor hERα cycles on the estrogen responsive *pS2* gene promoter with a period of approximately 20 minutes, which periodically prevents association of pol II and initiation of transcription [30]. Less regular oscillations with the average period of 5 hours were detected after DNA damage in human tumour-derived cells transfected with p53-CFP [31]. A more complicated form of gene expression pulsing is represented by circadian rhythms, which requires also external signals for maintaining or shifting the phase of the biological clock [32,33].

Transcriptional fluctuation should be distinguished from the transcriptional cycle (initiation, elongation, termination and recycling of the RNA polymerases) [34], which may, however, sometimes interfere with periodicity of transcription since the period of the polymerase recycling is usually comparable to the elongation time [1].

Even this brief sketch suffices to show that the phenomenon of transcriptional fluctuation, which has become known only recently, is a common feature of gene expression.

## 2. Detection of discontinuous transcription

Discontinuous transcription can be studied *in vivo* as well as after fixation/lysation of the cells (Table 1). One important approach is based on the transcription arrest by a proper inhibitor, e.g. α–amanitin, with a subsequent fixation or lysis of the cells at various time points after release from the block [17,21]. Subsequently, quantitative PCR (qPCR) and other quantitative methods are used to analyze the transcriptional fluctuation in a cell population. Nevertheless, some significant events may be overlooked, when the differences among the individual cells are averaged.

**Table 1.** Methods used for detection of transcriptional fluctuations.

	Method	Advantages	Disadvantages
**In live cells**	1. Gene trap with a luciferase reporter [6,20]	Measuring bursting frequency directly	Kinetic characteristics are assessed only at the level of the protein expression; abortive transcriptions and quick fluctuations escape observation
	2. Visualization of transcripts by bacteriophage fluorescent coat proteins (e.g. MS2) [17-19,42-48]	Measuring the chief kinetic parameters directly	Quantification requires additional assumptions
	3. Variant of (2) with inducible transcription [49]	As in (2), plus simultaneous assessment of the post-splicing movement of a single mRNA	As in (2), plus limitations attending the additional complexity
	4. Direct measurement of transcription by microspectrometry [23]	Requires no invasive treatment, can continue for hours	So far for pol I only, low resolution
	5. Measuring intensity of RNA polymerase signal *in situ* [25]	Relatively simple, applicable for individual genes or transcription factories	Indirect; kinetics of the enzyme and the transcription may differ significantly
**In fixed / lysed cells**	6. Transcription block and Release [17,21]	May include qPCR and other quantitative methods	Error of averaging; side effects of inhibitors
	7. smFISH [21,35-40]	Counting transcripts with high precision	Indirect, must be combined with transcription block and/or modelling
	8. Electron microscopy (chromatin spreads [2], isolated chromatin rings [41])	Visualization of individual transcription units	Indirect

Single molecule RNA fluorescence in situ hybridization (smFISH) is used for quantitative analyses of gene expression and nascent transcripts in a certain period of time at the single cell level [35–37]. The number of new RNA molecules may be determined with high precision by comparing the frequency and intensity of the fluorescence signal in the cell with a set of standard dilutions [38–40]. The results of such quantification alone provide indirect, but valuable information for modeling the expression kinetics in a cell population or tissue, when the studied gene is supposed to be transcriptionally active in all the cells [21]. Indirect data, e.g. the kinetics of homologous chromatids, can be obtained by visualization of nascent transcripts upon the chromatin spreads [2]. Isolation of chromatin rings followed by psoralen treatment and denaturation allowed to observe distribution of nucleosomes at the promoter of a yeast gene [41]. By calculating the frequencies of various configurations on electron microscopic preparations the authors connected the fluctuations of the gene expression with the nucleosome dynamics.

To observe the transcriptional activity *in vivo,* cells are transfected with various constructs providing a fluorescent signal that corresponds to the expression of a particular gene. In the method based on a gene trap strategy a luciferase gene is inserted under the control of endogenous regulatory sequences [6]. Since both the luciferase protein and its mRNA are short-lived, the method allows to calculate the key parameters of the transcriptional kinetics such as the rates of switching the promoter on and off [6,20].

The MS2 and PP7 based labelling methods, have become widely popular after the work of Chubb *et al.* on myxamoeba *Dictyostelium* [17]. These methods employ fluorescently labelled coat proteins of bacteriophages [17,42,43]. The MS2 or PP7 RNA transcripts form stem loop secondary structures that are recognized by the GFP-tagged coat protein. The repetitive nature of the MS2 or PP7 sequences results in binding of several GFP-tagged coat proteins to the same mRNA and consequently in a high single molecule sensitivity. GFP molecular equivalents of solubilized fluorophores (MESF) can be used for estimating the number of RNA molecules in each spot [44]. The fluctuations of the spot intensity in the transfected cells reflect the dynamics of the transcription. Imaging directly the transcript instead of its protein product is an obvious advantage of this strategy. It allows not only to monitor the intensity of a gene expression, but also to analyze separately the contributions of initiation, elongation, and termination [18,19,45–48]. In a more complicated system used by Shav-Tal *et al.* [49], a genetic locus, its transcribed mRNAs, as well as the translated protein were visualized in the cells expressing the cyan or red fluorescent protein fused to the lac repressor protein. In this system the lac repressor protein labels the genomic locus, the MS2-yellow fluorescent protein labels nascent transcripts and pTet-On controls transcriptional induction. The system allows to follow the production of a transcript as well as the subsequent movement of single messenger RNA – protein complexes in the cell nucleus.

Although the described FISH and GFP based methods have provided most of the data about discontinuous transcription, both approaches have their shortcomings. The entire kinetics cannot be deduced from the state of fixed cells and transcription inhibitors may produce uncontrollable side effects. Moreover, transfection of cells with two different complex constructs can also introduce some inaccuracy [40]. Single molecules are efficiently detected by the MS2 technique in bacteria [4], but in the case of eukaryotes it is still not clear how the continuous fluctuations of intensity in a fluorescent spot reflect the intermittent production of RNA molecules [40,50]. Therefore, endeavours were made to correlate the alternative approaches. Dar *et al.* compared the data on the activity of an HIV viral promoter obtained by the RNA FISH and MS2 based strategy, and found a general concordance of the two methods in terms of the numbers of molecules produced in one transcription burst [51].

## 3. Causes and modulators of discontinuous transcription

**Figure 2.**
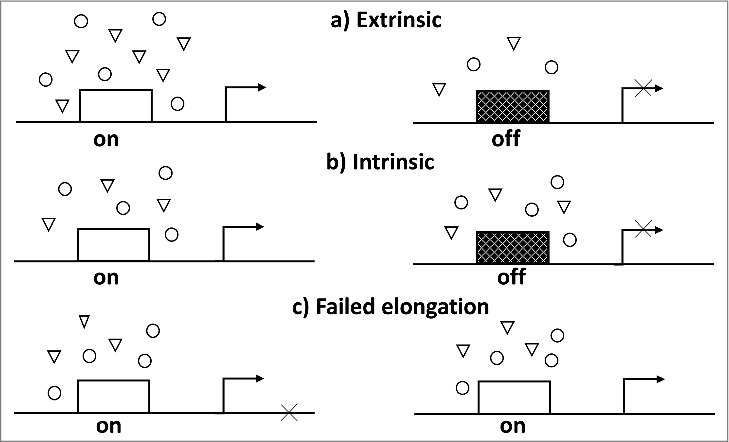
Probable causes of discontinuous transcription. Each diagram represents a DNA locus (straight line) with its promoter (rectangle), transcription start site (bent arrow), and various components of the transcription machinery (circles and triangles). a) Extrinsic causes: the promoter switches between the active (on) and inactive (off, cross-hatched box) states depending on the level of one or several factors in its environment; b) Intrinsic causes: inherently discontinuous activity of the promoter, it may be inactivated or reactivated even when the contents of its environment do not change; c) Transcription is paused at the stage of elongation (e.g. as a result of an error) and then it is resumed (e.g. after a correction); the status of the promoter does not change.

Transcription fluctuations can be divided into intrinsic and extrinsic based on their assumed causes (Figure 2). Variations in the levels of pol II machinery components, free nucleotides and other important factors are extrinsic factors that can be responsible for the pulsatile activity of the promoter [52–54]. Evidence for the extrinsic causes of the discontinuity is provided by the cases in which different genes are transcribed synchronously. Chromatin spreads reveal fiber-free gaps in the transcription units; when such gaps occupy symmetrical positions on the sister chromatid, it suggests a simultaneous silencing in the homologous genes [2]. The transfection experiments, in which two reporter genes were inserted into mutually remote regions of the genome, showed no synchrony, whereas when the two genes were located near each other, their expression was synchronous [55]. The transcriptional activity of the ribosomal genes from different nucleoli also seems to be synchronous [23]. The fluctuations can be caused by physiological signals such as hormone stimulation of glucocorticoid receptor [40,56]. Schoenfelder *et al.* suggested that periodicity of gene expression may result from periodical association of different chromosomal loci in the transcription factory [57].

Dynamic characteristics of transcriptional fluctuation can be altered by factors extrinsic in respect to the given gene and its promoter [1,53,58–61]. This most likely also includes a cooperation of closely positioned transcription units [6,22]. The chromosomal position of the genes undoubtedly has an impact on the kinetic of RNA synthesis. The same reporters were transcribed synchronously when inserted in the same locus and asynchronously when integrated into different loci [14,62]. In those cases, when several promoters are activated by one enhancer, the transcription activity of the respective genes may also correlate [63].

Transcriptional oscillation can be also caused by intrinsic factors, that is an inherently discontinuous activity of the promoter. Such behaviour is often referred to as “intrinsic noise” [52–54,58,64–66]. However, the stochastic nature implied by this term is difficult to reveal. Statistical estimation of the process by a standard random model would be insufficient for such demonstration. Similarly, if distribution of the intervals between certain bases in a DNA sequence well fits a stochastic model, it does not signify that the given sequence is a mere noise. Therefore, the terms “noise,” “random,” “stochastic,” widely used in the studies of discontinuous gene expression, are more or less provisional.

The random telegraph model has been used as a relatively simple way to describe the intrinsic fluctuations [14,6,67]. According to this model, a gene can be in one of two states: “on” or “off”. Transition between these two states is randomly determined and may be expressed by two constants that indicate the rates or probabilities of switching from the silent to the active status and back. The model predicts that the periods during which a gene will stay inactive follows exponential distribution, so that the shortest intervals of silence will appear most frequently. Some data of smFISH in isogenic population of both prokaryotic and mammalian cells fit into this scheme [14,21,68]. The distribution of RNA molecules per cell in such a simple model can be described by the Poisson statistics, which implies that the events (productions of the individual molecules) are mutually independent, that they occur with a constant frequency, and that the probability of the RNA production in a short interval is proportional to the length of this interval. Single-RNA counting in yeasts is consistent with the model based upon this statistics [21]. However, the simplest model has been found insufficient in many reports [40]. The distribution profiles of RNA molecules often appeared too broad to fit the Poisson statistics [53,60,61]. Besides, the duration of the silent periods often shows asymmetrical distribution, suggesting a non-equilibrium process [69]. An alternative is represented by the bursting model, which assumes that brief periods of high expression intensity (frequent production) are alternated by long periods of insignificantly low intensity (rare or no production) [50,70–72]. The size of the bursts represents the average number of transcripts.

Since numerous specific interactions precede the initiation of RNA synthesis, it has been suggested that in the silent state that follows each transcription burst at least one additional period (the refractory period) is needed before the gene may be switched on again [6,73]. In other words, a memory of the system, i.e. its dependence on the past, should be taken into consideration, since the length of time that has passed in the inactive state affects the length of time to be spent until the next active phase [74]. Such dynamics is already incompatible with the mathematical models based on the analysis of single cell RNA counting [14]. In contrast, a transition from the burst to silence seems to involve no refractory period and no memory: turning gene off is much easier than turning it on [1].

An even more complicated model of the bursting gene expression was suggested in the recent study of the actin gene in *Dictyostelium* [50]. The authors postulated a wide „spectrum“ of states with variable rates or probabilities of initiation. These states correspond to a potentially large number of coordinated steps preceding the actual transcription. This view is supported by the data of qPCR or chromatin immunoprecipitation (ChIP) experiments conducted at different terms after the induction of a gene expression [1,74,75]. For example, it was shown that after the addition of a stimulant (estrogen) the promoter of the estrogen target gene is bound sequentially by trefoil factor 1 and other factors including histone methyltransferases, histone acetyltransferases, then general transcription factors, and finally Pol II [75–77].

The discontinuous nature of transcription is apparently related to a repetition in the higher-order levels of chromatin structure involving substantial nucleosome-nucleosome interactions [1,40,60,61,78], which has been described as “breathing“ [55]. Each act of transcriptional bursting seems to require a separate act of chromatin decondensation, arrangement of transcription bubble, nucleosome opening, binding of transcription factors to promoter and enhancer, assembly of polymerase machinery, isomerisation (conformational changes of DNA and associated proteins), escape from promoter, and the last termination of the series. These processes are regulated by interplay of transcription factors [79,80], activity of chromatin remodelling complexes [1,61,62,81], formation of gene loops and pre-initiation complex assembly [21,60], disassembly and position-specific sliding of the nucleosomes [40]. Promoter sequence plays a significant role in genesis of the fluctuations. **S**pecific DNA features such as different numbers of CCAAT boxes may strongly influence the character of transcriptional kinetics [6,21,82]. The “phase separation model” suggests that super-enhancers can maintain stable bursting expression of several genes with high frequency [83].

Another plausible cause of the transcription discontinuity can be found downstream of the promoter. The so-called promoter proximal posing [84,85], which occurs after initiation when RNA polymerase has passed 20 – 50 bp, can significantly affect the pattern of gene expression. The phenomenon is probably not rare. The studies in *Drosophila* and mammalian cells show that pol II accumulates at the 5’ end of 20–30% genes including actively transcribed loci. The pause may serve for coordination of transcription with RNA processing. It is not clear how the RNA synthesis is restarted after the arrest, but it was reported that the proto-oncogene c-Myc plays a direct role in the pause release [86].

Elongation may be also stalled at the 3’end, close to the termination site, in other parts of the gene, and sometimes in a sequence dependent manner [87–89]. Occasionally, the pauses are followed by the reverse translocation of the RNA polymerase, a process known as backtracking, which is caused by errors in the nucleotide incorporation or by the formation of a weak RNA-DNA hybrid [90–92]. All these events prevent continuous transcription.

Thus, discontinuous gene expression may have numerous causes, but it seems probable that the dynamic pattern of transcription depends on a few dominant factors.

## 4. Kinetic characteristics of discontinuous transcription

Short periods of intensive transcription frequently alternate with longer silent intervals, which justifies the usage of the term ”bursting” [6]. However, the kinetic characteristics or parameters of the transcriptional fluctuation vary significantly and can be regulated separately or in combinations [1,6,22]. Special controls exist at the level of recruitment, initiation, pausing, and elongation of the transcription by RNA polymerase II [85]. The transcription dynamics therefore reflects the underlying regulatory principles of gene expression [93].

The following three kinetic parameters seem to be the most important ones: the burst size, the coefficient of variation and the frequency. The burst size, which corresponds to the number of RNA molecules produced in a burst, is limited by the rate of transcription: if one pol II complex occupies about 60 bp of DNA strand, and passes roughly the same distance in a second, it cannot produce more than one transcript in a second [6]. Some genes, e.g. an immunoglobulin gene in plasmacytoma cells [94], seem to be able to approach this speed limit, but others are transcribed rarely. The average burst size of the HIV-1 long terminal repeat ranges from 2 to 5 mRNA molecules as determined from the smFISH data and from the GFP signal *in vivo* [51]. The average burst size can be controlled by varying the amount of transcriptional activators in the cell [14].

Another important parameter of transcriptional fluctuations is the coefficient of variation (CV), i.e. the standard deviation of the gene expression level divided by its mean value. In mammalian cells the mean expression is not determined by CV, but by the burst size and the burst frequency [63,95]. Numerous quantitative data suggest that the CV inversely correlates with the mean expression [50]. Thus, after adding the inflammatory cytokine TNFα, the expression of the *HIV LTR* gene increased, but the CV decreased [15]. Such data are remarkable, since they imply that the burst size tends to remain constant and the expression level depends mainly on the frequency of the bursts.

The frequency is a highly variable parameter of bursting. In different genes the average periods of fluctuation (intervals between the bursts) vary from a few minutes to hours [6,17,50,73]. In many cases burst periods last a few minutes or less [50], but Muramoto *et al.* observed both short (from 2.5 min) and long (up to 27.5 min) pulses in the expression of the *act5* gene in *Dictyostelium* [22]. Since transcriptional fluctuation is usually irregular, spectral analysis of the time series may be useful in its study. Thus periodograms for the fluctuations of pol I signal on the ribosomal genes revealed several spectral components probably reflecting a complex fluctuation pattern of transcription [25].

Some of the kinetic parameters that are not directly observed may be inferred from other data. The number of nascent mRNA molecules can be used as a proxy for the polymerase occupancy; knowing the length of the gene, the polymerase speed (assumed to be approximately 30 bp/sec), and the polymerase occupancy, one can calculate the transcription rate. All these parameters vary significantly in isogenic cells [7,78]. Using minimal models of promoter cycles, Zoller *et al.* were able to identify two other parameters: the number and duration of inactive states [78]. The authors inserted various promoters or trapped the endogenous promoters in the murine NIH 3T3 cells and by measuring the luciferase luminescence with 5 min intervals they found from 1 to 7 states of inactivity with the duration of 6 to 14 min. The timing suggested that the dynamics of histone modifications rather than the interaction of transcription factors with DNA was responsible for the variety [78].

Thus, the kinetic characteristics and especially the burst frequency are highly variable which should be carefully considered in the studies of transcriptional fluctuations.

## 5. Physiological significance of discontinuous transcription

In unicellular organisms, especially in bacteria and yeasts, burst-like gene expression causes heterogeneity in populations, since variation in mRNA levels is easily translated into heterogeneity of protein levels and phenotype diversity including adaptation to unfavourable factors [4,68,96–101]. Thus, the frequency of transcriptional bursts of the *cI* gene regulates transition between the lysogenic and lytic phases in the bacteria infected by the lambda phage [102]. In some cases resistance of bacteria to antibiotics [103], or that of tumour cells to chemotherapy [104,105], may be attributable to non-genetic differences, among which the phase of transcription fluctuation is perhaps the most common. Advantages of the dynamic variability of the cells improve the chances of a clonal population to adapt to variable conditions [100,106].

In multicellular organisms variations of protein levels are relatively low [20,107]. However, differentiation of embryonic stem cells is associated with extensive changes in gene expression and fluctuations in gene expression at a certain stage of development may alter the post-mitotic fate. Thus, discontinuous transcription becomes a source of cell diversity [108]. Differentiation of retina [109], neurons [110], myoblasts [111], haematopoetic cells [10], intestinal cells [70], seems to involve a cell fate choice based on the bursting gene expression. Transcriptional fluctuations are proposed to be a major driver of the spontaneous heterogeneity in gene expression, which in turn drives the diversity of cell behaviour in changing environmental conditions, differentiation, and disease [70,100].

Under certain conditions fluctuations may be detrimental to the cell homeostasis and they have to be suppressed [112,113]. The schemes of such suppression through feedback loops are proposed for bacteria and yeast [114].

The remarkable ubiquity of transcriptional fluctuations suggests that their role in regulatory processes in the cell are likely to be enormous. For one thing, pulsing is likely to cause other pulsing. Thus, RNA processing, which is closely linked to the RNA synthesis, may also occur in pulses [14,115]. Extensive data about the connection between the synthesis and processing kinetics of a single RNA may be obtained by the “fluctuation analysis” based on computing and interpreting cross-correlation functions [48]. On the other hand, burst size measurements suggest that enhancers and suppressors of the fluctuations modulate the state of the cell, e.g. awakening the HIV virus from latency [51]. Hardly anything is known about the transcription kinetics of long non-coding RNAs, which are also likely to be transcribed in pulses and their kinetics may be essential for their regulatory role in the cell nucleus.

Perhaps the term “burst” was coined with regard to neurobiology, where it is applied to a pattern of action potential in an axon [41]. A tendency of the burst size to stability suggests that transcriptional fluctuation, like a neuronal signal, can be resistant to noise and serve as a channel of information [15], though, for the present, it is difficult to tell how far the analogy may go.

## 6. Conclusion

The research on discontinuous transcription has been intensified in the last years and this rising interest in the phenomenon reflects a recognition of its biological relevance. There seem to be two essential problems to be explored: 1) the nature of the local processes leading to pausing and restarting of transcription; 2) the interaction of various transcriptional and non-transcriptional fluctuations in the cell. Alternative approaches and new methods will most likely be needed to resolve these problems. The suggested lists of requirements include improved procedures for arresting transcription; establishing the order of events at the promoter; understanding whether transcriptional fluctuations are stochastic or specifically regulated [116]; methods for higher throughput monitoring, which would allow to study expression dynamics of multiple genes [117]. With these and other innovations considerable findings potentially pointing to a new level of gene regulation may be made in the near future.
